# Integrating Free Amino Acid Profiles with Flavoromics to Characterize the Flavor Characteristics of Different *Morchella* Species

**DOI:** 10.3390/foods15081424

**Published:** 2026-04-19

**Authors:** Jie Li, Jinyan Liu, Yixin Li, Zihan Gao, Le Wang, Qian Song, Ying Ye, Jian Liang

**Affiliations:** 1 College of Agriculture and Animal Husbandry, Qinghai University, Xining 810016, China; xli6818@163.com (J.L.); liujinyan0111@163.com (J.L.); 2 State Key Laboratory of Plateau Ecology and Agriculture, Xining 810016, China; 15737724420@163.com (Y.L.); cngaozihan@163.com (Z.G.); wangleqhu@163.com (L.W.); 2023990003@qhu.edu.cn (Q.S.)

**Keywords:** *Morchella*, free amino acids, volatile organic compounds, TAV, rOAV

## Abstract

This study presents a comprehensive flavour profile analysis of 12 *Morchella* samples (5 cultivated and 7 wild species) collected from diverse regions across China. The contents of free amino acids and volatile organic compounds were determined using UHPLC-QE-HRMS and HS-SPME-GC-MS. Flavour contribution was assessed by calculating taste activity values (TAVs) and relative odor activity values (rOAVs), and the influence of environmental factors on flavour compound accumulation was further explored. The findings indicated that cultivated *Morchella* exhibited pronounced fruity, floral, sweet, and mushroom-like notes (e.g., 1-octen-3-one, beta-damascone, and 1-(2-aminophenyl)ethanone), rendering them suitable for fresh consumption. In contrast, wild *Morchella* exhibited higher levels of herbaceous and smoky aroma compounds (e.g., (E,Z)-2,6-nonadienal, benzenemethanethiol, and non-8-enal), suggesting potential for premium product development. Correlation analysis revealed metabolic associations between taste-active amino acids and key volatile organic compounds via intermediates of the lipoxygenase pathway and the tricarboxylic acid cycle. Furthermore, environmental parameters including elevation, annual precipitation, and solar radiation were found to significantly influence the accumulation of flavour-related metabolites. These findings provide insights into the chemical basis underlying the flavour diversity of *Morchella* and offer a theoretical foundation for species identification, flavour-directed breeding, and differentiated product development.

## 1. Introduction

*Morchella*, a genus of rare and valuable edible and medicinal macro-fungi, belongs to the phylum *Ascomycota*, class *Pezizomycetes*, order *Pezizales*, and family *Morchellaceae* [1]. In China, *Morchella* spp. are collectively known as one of the four most celebrated edible fungi, alongside *Tricholoma matsutake*, *Termitomyces*, and *Dictyophora indusiata*, owing to their delicious taste, fleshy texture, and rich nutritional profile comprising proteins, vitamins, polysaccharides, and lipids [2], as well as their associated physiological functions including antioxidant, antitumor, anti-inflammatory, and immunomodulatory activities [3,4,5,6]. The unique flavor of morels is determined by the combined contribution of both volatile and non-volatile compounds [7]. The composition and relative abundance of these compounds are critical factors in evaluating morel quality, directly influencing consumer preference, palatability for fresh consumption, and suitability for processing [8]. Currently, the primary cultivated *Morchella* species in China is *M. sextelata* [9].

Free amino acids, as crucial non-volatile flavor compounds in *Morchella*, significantly influence the taste and mouthfeel upon consumption [10]. Existing research indicates that morels are rich in amino acids essential for the human body, among which Glu and Asp are the primary taste-active amino acids contributing to umami perception. These compounds can synergistically enhance the overall taste experience when combined with flavor nucleotides such as inosine monophosphate and guanosine monophosphate. Furthermore, studies have revealed that the content and composition of taste-active amino acids in morels vary considerably depending on factors such as species, geographical origin, and drying methods [7,11,12]. Stojek et al. also found that the protein composition and amino acid content of wild fungi are closely related to their growth environment, including temperature, humidity, and soil properties; moreover, the environmental impact on wild fungi also varies by species [13]. Notably, serving as precursors for volatile organic compounds (VOCs), amino acids can indirectly influence the generation of volatile flavor substances through Strecker degradation and the Maillard reaction [14,15]. However, current research on free amino acids in *Morchella* has largely focused on the nutritional quality assessment and comparison of a limited number of cultivated species. There is a lack of systematic classification and comparative studies on the composition of taste-active amino acids and their associated taste profiles across different *Morchella* species, and comparative investigations between cultivated and wild species are particularly scarce.

Volatile flavor compounds are central to determining the unique aroma of *Morchella*, and their composition, concentration, and sensory thresholds collectively shape the characteristic flavor profiles of different morel species [16]. The accumulation and expression of these flavor compounds are influenced by a combination of factors, including species, genetic background, growth substrate, environmental conditions, and post-harvest processing methods [6,9,17,18]. To date, a diverse array of VOCs has been identified in morels, encompassing ketones, aldehydes, alcohols, esters, and sulfur-containing compounds [19,20]. Among these, 1-Octen-3-ol, commonly known as “mushroom alcohol,” serves as a signature compound for the typical fungal mushroom-like odor and is generated from unsaturated fatty acids such as linolenic and linoleic acid via fatty acid metabolism [21]. Meanwhile, sulfur-containing compounds such as dimethyl sulfide and methylthiopropanal impart a pronounced meat-like aroma to edible fungi, with their formation linked to the metabolism of sulfur-containing amino acids [22]. This clearly suggests a metabolic interconnection between amino acid metabolism and the generation of volatile flavor compounds. However, studies integrating amino acid profiles with volatile compound analysis in *Morchella* remain limited. The specific mechanisms by which taste-active substances influence the formation of volatile aroma compounds have yet to be fully elucidated. Furthermore, systematic comparative investigations examining both amino acid and volatile flavor profiles across different cultivated and wild *Morchella* species are notably insufficient.

Therefore, this study investigated 12 *Morchella* samples from different species and geographical origins, comprising five cultivated species and seven wild species. The amino acid profiles and volatile compound compositions of these different *Morchella* species were determined and comprehensively analyzed using ultra-high-performance liquid chromatography coupled with tandem high-resolution Orbitrap mass spectrometry (UHPLC-HRMS) and headspace solid-phase microextraction coupled with gas chromatography-mass spectrometry (HS-SPME-GC-MS). The flavor contributions of taste-active amino acids and VOCs were evaluated using taste activity value (TAV) and relative odor activity value (rOAV) analysis, respectively, thereby establishing a comprehensive flavor evaluation system for the fruiting bodies of different *Morchella* species. This study aims to scientifically evaluate and elucidate the material basis of flavor and the inter-varietal differences among *Morchella* species, systematically analyze their suitability for differentiated product development and processing, and provide a theoretical foundation for the rational development and innovative utilization of *Morchella*.

## 2. Materials and Methods

### 2.1. Materials and Reagents

Sample Collection: A total of 12 *Morchella* samples from different species and regions across China were selected for this study, with each sample representing an independent biological replicate. Each replicate was composed of more than ten fruiting bodies of the same species, collected at the mature stage under identical cultivation conditions. The sampled portion was the whole fruiting body (including the pileus and stipe). The maturity of the fruiting bodies was determined based on the following criteria: the ascocarps no longer increased in size, the stipes appeared pale yellow, the ridges and pits on the pileus exhibited clear contours with the color shifting from brownish-yellow to dark brown, and the flesh was thick and elastic. Among these, five cultivated *Morchella* samples were collected from Gangcha County (XHQH-2, XHQH-6), Jianzha County (JZ), Tianjun County (TJ), and Ping’an District (PA) in Qinghai Province. Seven wild *Morchella* samples were collected from Nyingchi City, Tibet Autonomous Region (XZ); the Qinling Mountains, Shaanxi Province (QL); the Changbai Mountains, Jilin Province (CBS); Shangri-La City, Yunnan Province (XGLL); and Huzhu Tu Autonomous County (HZ), and Golog Tibetan Autonomous Prefecture (GL) in Qinghai Province; Gannan Tibetan Autonomous Prefecture (GN) in Gansu Province. The geographical and environmental conditions of the collection sites for each *Morchella* species are presented in Table 1. The collected *Morchella* fruiting bodies were placed into preservation bags, then immersed in liquid nitrogen for 1 min to achieve rapid freezing, and then transferred to foam insulation boxes containing sufficient dry ice for transport to the laboratory. The samples were pre-frozen at −80 °C for 4 h, followed by freeze-drying in a CoolSafe 55-9 freeze dryer (ScanVac, LaboGene A/S, Allerød, Denmark) for 48 h, with a cryo-trap temperature of −55 °C and a chamber pressure of 0.001 mBar. After freeze-drying, the water content of the samples was approximately 3%. The freeze-dried samples were pulverized using a BJ-300A high-speed grinder (Baijie Co., Ltd., Hangzhou, China). To avoid overheating, an intermittent grinding method was applied (30 s grinding followed by 60 s cooling to room temperature) and repeated until the samples passed through an 80-mesh sieve, with a total loss rate below 5%. The powder was stored in 25 mL centrifuge tubes (polypropylene, screw cap) and stored at −20 °C until further analysis.

Free amino acid standards: Cys–Cys, Asp, Glu, Pro, Ala, Thr, Gly, Ser, Arg, Lys, His, Val, Phe, Ile, Met, Trp, Leu, Tyr, Asn, γ-aminobutyric acid (GABA), Orn, Cit, 2,4-diaminobutyric acid (DABA), Hyp (4-hydroxy-L-proline), and Gln (purity ≥ 99%) were purchased from Sigma-Aldrich (St. Louis, MO, USA). All amino acid standards used in this study were of L-configuration, except for Gly.Sodium chloride (AR, ≥99.5%) and hydrochloric acid (AR, 36–38% (mass fraction)) were purchased from Sinopharm Chemical Reagent Co., Ltd. (Beijing, China). n-Hexane (GC, 98%) was purchased from Merck KGaA (Darmstadt, Germany). Methanol (GC, ≥99.9%), acetonitrile (GC, ≥99.9%), and formic acid (GC, 98%) were purchased from Shanghai ANPEL Experimental Technology Co., Ltd. (Shanghai, China). The derivatization reagents were AccQ•Tag Ultra Borate buffer and AccQ•Tag reagent (Waters Corporation, Milford, MA, USA).

### 2.2. Analysis of Amino Acid Composition and TAV

#### 2.2.1. Determination of Amino Acid Content

Free amino acid content in different *Morchella* samples was determined using ultra-high-performance liquid chromatography coupled with tandem high-resolution Orbitrap mass spectrometry (UHPLC-QE-HRMS, Thermo Fisher Scientific, Waltham, MA, USA). Accurately weighed samples were transferred to centrifuge tubes, and 1 mL of 0.1 M hydrochloric acid solution was added. After vortexing, the mixture was extracted at room temperature (approximately 25 °C) for 1 h, followed by centrifugation at 13,800× *g* for 10 min at 4 °C. The supernatant was collected and diluted accordingly. For derivatization, 10 μL of the sample solution was transferred to a derivatization tube, followed by the addition of 70 μL of AccQ•Tag Ultra Borate buffer and 20 μL of AccQ•Tag reagent. The mixture was vortexed, heated at 55 °C for 10 min, cooled to room temperature, and then subjected to UHPLC-QE-HRMS analysis.

Liquid Chromatography Conditions: Chromatographic separation was performed on a ChromCore 120 C18 column (100 × 4.6 mm, 3 μm). The mobile phase consisted of ultrapure water containing 0.1% (*v*/*v*) formic acid (A) and acetonitrile containing 0.1% (*v*/*v*) formic acid (B). The flow rate was 0.5 mL/min, the column temperature was maintained at 50 °C, and the injection volume was 1 μL. The gradient elution program was as follows, using a linear gradient between the time points: 0 min, A/B (95:5, *v*/*v*); 1.0 min, A/B (95:5, *v*/*v*); 3.5 min, A/B (60:40, *v*/*v*); 5.0 min, A/B (25:75, *v*/*v*); 8.0 min, A/B (20:80, *v*/*v*); 8.1 min, A/B (95:5, *v*/*v*); 10.0 min, A/B (95:5, *v*/*v*). Throughout the analysis, samples were maintained at 4 °C in the autosampler. Quality control (QC) samples (prepared by pooling equal volumes of supernatant from all samples) were inserted uniformly throughout the sample sequence to monitor system stability and ensure the reliability of the experimental data.

Mass Spectrometry Conditions: Mass spectrometric detection was performed using a Q Exactive Plus high-resolution mass spectrometer (Thermo Fisher Scientific, USA) equipped with an electrospray ionization (ESI) source operating in positive ion mode. The sheath gas flow rate was set at 4.0 L/min, the auxiliary gas flow rate at 1.0 L/min (both N_2_, purity 99.999%), the ion spray voltage at +3000 V, the ion source temperature at 350 °C, and the ion transfer tube temperature at 320 °C. Data acquisition was performed in Full MS scan mode at a resolution of 70,000 over a mass range of m/z 200–600 [23]. The amino acid analysis method has been validated in previous studies with good linearity (R^2^ > 0.99) and recovery rates [24].

#### 2.2.2. Classification of Amino Acids

Based on the sensory characteristics perceived by human taste receptors, the structural features of amino acid side chains, and their interactions with taste receptor cells, free amino acids were classified according to their taste properties [25,26,27]. Following the classification framework established in references [28,29], amino acids were categorized into four groups: umami amino acids (UAAs), namely Asp and Glu, which are characterized by their ability to activate the T1R1/T1R3 taste receptor; sweet amino acids (SAAs), including Pro, Ala, Thr, Gly, and Ser, typically interacting with the T1R2/T1R3 receptor; bitter amino acids (BAA), comprising Arg, Lys, His, Val, Phe, Ile, Met, Trp, and Leu, often associated with T2Rs receptor interactions; and other amino acids (OAAs), which encompass those with sour taste, tasteless properties, or ambiguous sensory characteristics, including Tyr, Asn, GABA, Orn, Cit, DABA, Hyp, and Gln.

#### 2.2.3. Analysis of TAV

Following the methods described in Refs. [30,31], the TAV was calculated to evaluate the taste contribution of taste-active amino acids. The calculation formula is as follows:

(1) $$TAV_{i}=\frac{C_{i}}{T_{i}}$$

where *TAV_i_* represents the taste activity value of taste-active amino acid *i*, *C_i_* is the concentration of taste-active amino acid *i* (mg/g), and *T_i_* is the taste threshold of amino acid *i* (mg/g).

### 2.3. Analysis of VOCs

#### 2.3.1. HS-SPME

A 500 mg of lyophilized (freeze-dried) powder of each *Morchella* sample was immediately transferred into a 20 mL headspace vial (Agilent, Palo Alto, CA, USA) containing 2 mL of saturated NaCl solution to inhibit enzymatic activity. Then, 20 µL of the internal standard solution (3-Hexanone-2,2,4,4-d4, 10 µg/mL) was added to the vial. The vial was sealed with a crimp cap equipped with a TFE-silicone headspace septum (Agilent). For SPME analysis, each vial was equilibrated at 60 °C for 5 min, followed by exposure of a 120 µm DVB/CWR/PDMS SPME fiber (DVB: Divinylbenzene, CWR: Carbon Wide Range, PDMS: Polydimethylsiloxane; Agilent, product no. 5191-5874) to the headspace of the sample for 15 min at 60 °C. It should be noted that this study did not conduct separate spike-and-recovery experiments for the freeze-drying and grinding steps to accurately evaluate their recovery rates, which constitutes one of the limitations of this study. In-depth optimization and validation will be performed on this aspect in subsequent research.

#### 2.3.2. Analysis of GC-MS

After sampling, the VOCs adsorbed on the SPME fiber coating were desorbed in the injection port of a gas chromatograph (Model 8890; Agilent) at 250 °C for 5 min. Identification and quantification of VOCs were performed using an Agilent 8890 gas chromatograph coupled with an Agilent 7000D mass spectrometer, equipped with a 30 m × 0.25 mm × 0.25 μm DB-5MS (5% phenyl-polymethylsiloxane) capillary column. High-purity helium (99.999%) was used as the carrier gas at a constant volumetric flow rate of 1.2 mL/min. The injector temperature was maintained at 250 °C. The column temperature program was set as follows: initially held at 40 °C for 3.5 min, then increased to 100 °C at a rate of 10 °C/min, followed by an increase to 180 °C at 7 °C/min, and finally raised to 280 °C at 25 °C/min and held for 5 min. Mass spectrometric acquisition was performed in electron ionization (EI) mode at 70 eV. The temperatures of the quadrupole mass analyzer, ion source, and transfer line were set to 150 °C, 230 °C, and 280 °C, respectively. The mass spectrometer was operated in selected ion monitoring (SIM) mode for analyte identification and quantification.

#### 2.3.3. Qualitative and Quantitative Analysis of Metabolites

The raw data obtained after mass spectrometry analysis were processed using MassHunter software (Version B.08.00). A self-built database (Maiwei, Wuhan, China) containing confirmed retention times (RT) as well as quantitative and qualitative ions was used for accurate scanning in SIM mode. For each compound, one quantitative ion and two to three qualitative ions were selected. All target ions in each group were detected in segments according to their elution order. A compound was identified if its retention time matched that of the reference standard and all selected ions were present in the background-subtracted mass spectrum of the sample. The quantitative ions were used for integration and calibration to enhance quantification accuracy. A semi-quantitative internal standard method using 3-Hexanone-2,2,4,4-d4 as the internal standard was employed to calculate the relative content of VOCs in different *Morchella* samples according to the relevant literature [32,33,34], using the following formula (this analytical method has been validated in previous studies and is widely used in comparative metabolomics analysis):

(2) $$X_{i}=\frac{V_{s}\times C_{s}}{M}\times \frac{I_{i}}{I_{s}}$$

In the equation, *X_i_* denotes the concentration of compound *i* in the test sample (μg/g); *V_S_* represents the volume of internal standard added (μL); *C_s_* indicates the concentration of the internal standard (μg/μL); *M* signifies the mass of the test sample (g); *I_s_* denotes the peak area of the internal standard; and *I_i_* represents the peak area of compound *i* in the test sample (peak area was used for quantification).

#### 2.3.4. Analysis of rOAV

The rOAV method was employed to screen and determine the key flavour compounds of 12 *Morchella* species. Generally, an rOAV ≥ 1 indicates that the compound directly contributes to the sample’s flavour profile. Referencing relevant literature [35,36], the rOAV analysis was conducted using the following formula:

(3) $$rOAV_{i}=\frac{C_{i}}{T_{i}}$$

In the formula, *rOAV_i_* denotes the relative odor *C_i_* represents the relative concentration of the compound (μg/g); *T_i_* indicates the threshold value of the compound (Threshold, μg/g).

### 2.4. Data Statistics and Analysis

A total of 12 *Morchella* species were included in this study, with each species regarded as an independent biological replicate. Samples from each species were analyzed independently in triplicate under identical conditions, serving as technical replicates. All data presented as mean ± standard deviation. Unsupervised PCA (principal component analysis) was performed by statistics function prcomp within R (Version 4.1.2; www.r-project.org/). The data was unit variance scaled before unsupervised PCA. Data were analysed using SPSS 27.0 software, with *p* < 0.05 indicating statistically significant differences. Graphs were generated using Origin 2021 and GraphPad Prism 9 software.

## 3. Results

### 3.1. Amino Acid Composition and TAV Analysis of Different Morchella Species

#### 3.1.1. Analysis of Free Amino Acid Composition and Content in Different Species of *Morchella*

**Figure 1:**
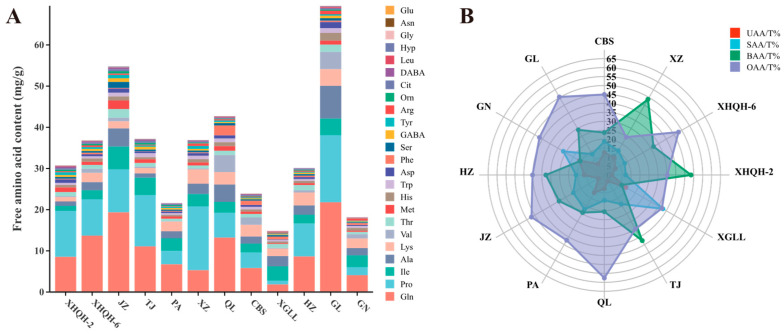
Stacked bar chart of free amino acid composition and content in different species of *Morchella*. (**A**) The detailed data on the content of free amino acids in each species are provided in Table S3; radar chart of taste characteristics of amino acids. (**B**) T represents total amino acid content.

Free amino acids, as important non-volatile flavor substances, are characterized by low taste thresholds and high taste intensity, with their composition and content serving as crucial determinants of *Morchella* flavor [37,38]. As shown in Figure 1A, a total of 19 proteinogenic amino acids and 6 non-proteinogenic amino acids were detected across the 12 different *Morchella* species (Cys-Cys was not detected). The total free amino acid content, expressed on a dry weight basis, ranged from 14.88 mg/g ± 1.7 mg/g to 69.51 mg/g ± 9.8 mg/g, with the ranking among species as follows: GL > JZ > QL > TJ > XZ > XHQH-6 > XHQH-2 > HZ > CBS > PA > GN > XGLL. Notably, the total amino acid content in GL was (4.66 ± 0.22)-fold higher than that in XGLL. This disparity is hypothesized to result from differences in *Morchella* species and their respective growth environments [37]. Furthermore, across all *Morchella* species, Gln, Arg, and Ala constituted the core amino acid components, accounting for 41.88% to 74.64% of the total amino acids. Among these, Gln serves as a central metabolite in human nitrogen metabolism and possesses multiple physiological functions [39,40]. Notably, the average amino acid content of the five cultivated *Morchella* species was 36.24 mg/g, while that of the seven wild species was 33.76 mg/g dry weight, showing an increasing trend in the cultivated group. This finding suggests that protracted domestication and cultivation may have selected for genotypes that are more conducive to amino acid accumulation, potentially enhancing their nutritional value [41].

#### 3.1.2. Analysis of Flavour-Contributing Amino Acids and TAV in Different Species of *Morchella*

When the TAV > 1, it indicates that the taste-active amino acid contributes significantly to the overall taste of the sample, with the magnitude of contribution being proportional to the TAV [42]. The taste attributes and TAVs of taste-active amino acids in different *Morchella* species are presented in Table 2. The results revealed that four amino acids—Glu (umami), Ala (sweet), Arg (bitter), and Asn (sour)—exhibited TAVs greater than 1 across all *Morchella* species, indicating that these taste-active amino acids universally contribute to the flavor profiles of all examined morels. In contrast, Gly had a TAV exceeding 1 only in JZ (TAV = 1.12 ± 0.24), suggesting that this sweet amino acid contributes significantly solely to the overall flavor of the JZ species. Radar charts were constructed based on the proportional contribution of each taste attribute (Figure 1B). The results demonstrated that although the proportion of OAA was relatively high across all species, ranging from 24.45% to 57.58%, TAV analysis indicated that its overall impact on flavor was limited. BAA proportions were notably higher in XHQH-2 (48.49%) and XZ (48.81%), with four bitter amino acids (Arg, Lys, His, and Val) exhibiting TAV >1 in both species, classifying XHQH-2 and XZ as bitterness-dominant flavor types. However, the taste activity of BAA is relatively low and can often be masked by other flavor components such as soluble sugars, sweet amino acids, and 5′-nucleotides, thereby mitigating bitter perception [6,43]. XGLL exhibited the highest proportion of SAA (37.76%). TAV analysis of taste-active amino acids in XGLL revealed that, with the exception of Arg (TAV = 1.82 ± 0.38), all BAA TAVs were below 1, while the combined TAVs for SAA and UAA both exceeded 5.50. This characterizes XGLL as exhibiting a sweet-umami synergistic flavor profile, imparting a clean, sweet, and round taste with enhanced palatability [44]. Similarly, GN, which had the highest UAA proportion (16.05%), was also classified as possessing a sweet-umami synergistic flavor based on TAV analysis. Furthermore, for GL, which contained the highest total amino acid content, UAA and BAA proportions were 7.08% and 29.12%, respectively, with combined TAVs exceeding 13.50. This indicates that GL exhibits a bitter-umami complex flavor, wherein subtle bitterness not only enriches the taste layering and perceived sweetness but also accentuates the characteristic “mountain delicacy” flavor of morels [45]. These distinctive compositions of taste-active amino acids constitute the material basis for the characteristic flavor profiles observed across different *Morchella* species and geographical origins.

### 3.2. Analysis of VOCs in Different Species of Morchella

#### 3.2.1. GC-MS Analysis of VOCs in Different Species of *Morchella*

**Figure 2:**
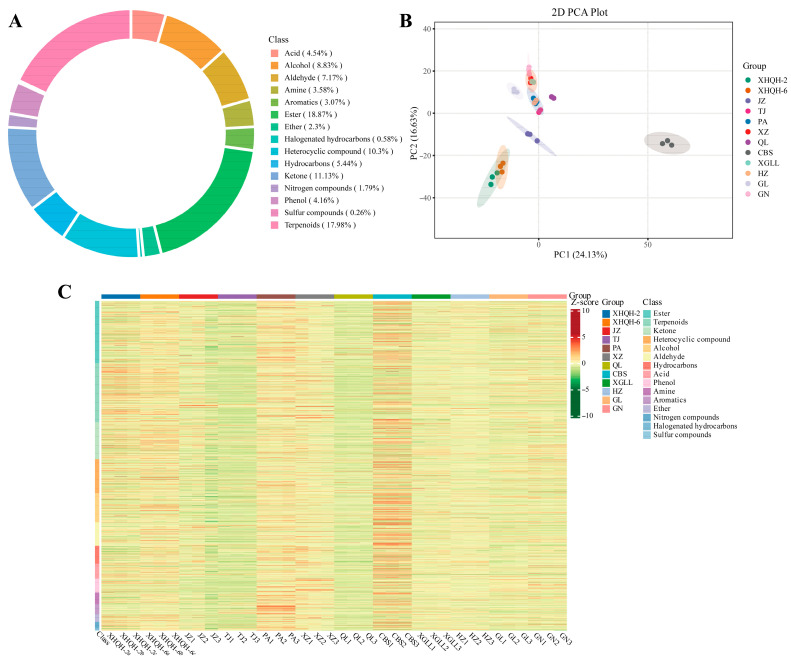
Circular chart showing the compositional categories of 1563 metabolites in different species of *Morchella* (**A**); PCA score plot (**B**); and overall hierarchical clustering analysis plot of samples (**C**).

VOCs composition analysis of fruiting bodies from 12 different *Morchella* strains was conducted using GC-MS, with results presented in Table S1. A total of 1563 compounds were detected, including 56 amines, 138 alcohols, 48 aromatic hydrocarbons, 65 phenols, 28 nitrogen-containing compounds, 4 sulphur-containing compounds, 9 halogenated hydrocarbons, 36 ethers, 112 aldehydes, 71 acids, 281 terpenes, 85 hydrocarbons, 174 ketones, 161 heterocyclic compounds, and 295 esters. The relative content of each VOCs class was calculated as the ratio of the peak area of the compounds in that class to the peak area of the internal standard, and the relative abundance of each class was expressed as the percentage of its relative content to the total relative content of all classes. Among these, esters (18.87%) and terpenes (17.98%) constitute the two most abundant VOC categories, collectively accounting for 36.85% and forming the flavour foundation of *Morchella*. Ketones (11.13%) and heterocyclic compounds (10.30%) form the second tier, totalling 21.43%. Other compounds including alcohols (8.83%), aldehydes (7.17%), hydrocarbons (5.44%), and acids (4.54%), though less abundant, also contribute to and refine the overall flavour profile of morels (Figure 2A) [7,46].

PCA was performed to provide a preliminary understanding of the overall metabolic differences among different *Morchella* species and the degree of variability within samples of the same species [47]. The PCA was performed based on the relative contents of all identified volatile flavor compounds across all samples (peak area normalization method). The PCA score plot (Figure 2B) revealed that the first two principal components accounted for 40.76% of the total variance (PC1 = 24.13%, PC2 = 16.63%). Although the cumulative contribution rate was less than half of the total variance, the score plot still clearly illustrated the separation trends among the different *Morchella* species. Furthermore, the biological replicates of the same *Morchella* sample clustered closely together, demonstrating good reproducibility and the stability and reliability of the experimental data [48]. Notably, CBS was completely separated from all other species along the PC1 axis, suggesting that its flavor metabolic profile is markedly distinct from the others, exhibiting a strong varietal specificity. XHQH-2 and XHQH-6 clustered tightly together in the negative region of both PC1 and PC2, and were clearly separated from the other species, indicating a high degree of similarity in their flavor metabolic characteristics, which also differ from those of the other species. The remaining species were dispersed throughout the plot, exhibiting some overlapping distribution, suggesting that while their flavor metabolic profiles share certain commonalities, distinct differences also exist. The hierarchical clustering analysis heatmap of the samples (Figure 2C) showed a high degree of concordance with the PCA results. Clear clustering branches were observed among different species, with CBS forming a distinct individual cluster and XHQH-2 preferentially clustering with XHQH-6. This clustering pattern aligns with the separation observed in the PCA and further substantiates the decisive role of varietal factors in shaping the characteristic flavor metabolic profiles.

#### 3.2.2. rOAV Analysis of VOCs in Different *Morchella* Species

The concentration of VOCs alone does not correlate with their contribution to flavor. The rOAV, which integrates VOCs concentration with its sensory threshold, quantifies the contribution of individual VOCs to the overall flavor and enables the identification of key flavor compounds in food [49]. A higher rOAV indicates a greater contribution of the corresponding VOCs to the characteristic *Morchella* flavor [50]. In this study, a total of 169 VOCs with rOAV ≥ 1 were identified. After eliminating those with no described aroma, 136 key VOCs were ultimately characterized, as detailed in Table S2. Among these, 15 VOCs with rOAV > 1000 were identified as core contributors to *Morchella* flavor (Table 3). These key VOCs comprised ketones (6 compounds), aldehydes (4), heterocyclic compounds (2), alcohols (1), terpenes (1), and esters (1). Collectively, they delineate the primary flavor characteristics of *Morchella* as predominantly mushroom-like and earthy, complemented by nutty and roasty notes, accompanied by subtle sweet fruity and fresh grassy and cucumber-like nuances. Furthermore, trace amounts of sulfides and alliaceous notes contribute to the overall flavor complexity of morels [9]. In the present study, 3(2H)-Furanone, dihydro-2-methyl-, characterized by sweet, buttery, and nutty notes, exhibited the highest rOAV across all *Morchella* species (ranging from 2.65 × 10^5^ to 9.01 × 10^5^), attributable to its relatively low odor threshold (5 × 10^−6^ μg/g). As a ubiquitous basal flavor compound present in all *Morchella* species, it provides a sweet and nutty base note that underpins the characteristic aroma profile. Notably, in the CBS species, several compounds exhibited markedly higher rOAVs compared to other species, including 3-Octen-2-one, (E,Z)-2,6-Nonadienal, (5Z)-Octa-1,5-dien-3-one, and 2,4-Nonadienal. Particularly striking was 2,4-Nonadienal, which imparts fatty, green, and cucumber-like notes and displayed an exceptionally high rOAV ((2.13 ± 0.13) × 10^5^). Specifically, its rOAV in CBS was (88.3 ± 4.5)-, (73.1 ± 5.7)-, (90 ± 11)-, (121.0 ± 6.4)-, (59.8 ± 3.9)-, (173.1 ± 6.3)-, (29.8 ± 3.0)-, (145 ± 13)-, (134 ± 10)-, (126.8 ± 9.9)-, and (128 ± 12)-fold higher than that in XHQH-2, XHQH-6, JZ, TJ, PA, XZ, QL, XGLL, HZ, GL, and GN, respectively. This pronounced difference may be attributed to the unique ecological environment of the Changbai Mountains (CBS), a region characterized by Cenozoic volcanic landforms with predominantly volcanic ash soils [51]. This edaphic environment differs substantially from the growth substrates of other *Morchella* species examined. Previous studies have demonstrated that certain VOCs in morels exhibit habitat-specificity [6], and the distinctive flavor profile of CBS likely reflects this phenomenon. This finding aligns with the PCA results, wherein CBS formed a completely separate cluster from all other species. Furthermore, *β*-Damascone, which imparts fruity, floral, and tobacco-like notes, exhibited substantially higher rOAVs in XHQH-2 ((8.45 ± 0.97) × 10^4^) and XHQH-6 ((5.9 ± 1.5) × 10^4^) compared to species from other regions. As a degradation product of carotenoids, the biosynthesis of *β*-Damascone is catalyzed by carotenoid cleavage dioxygenases (CCDs). Existing research suggests that low temperature, drought, and strong ultraviolet radiation promote CCDs expression, potentially explaining the high accumulation of *β*-Damascone observed in XHQH-2 and XHQH-6 [52]. Collectively, these findings demonstrate that the formation of characteristic flavors in different *Morchella* species is driven by the synergistic interaction of multiple environmental factors, resulting in the distinctive metabolic profiles observed across species and geographical origins.

Notably, the rOAV contributions of VOCs differed substantially between the five cultivated and seven wild *Morchella* species. Among the average rOAVs of VOCs in the five cultivated species, compounds such as 1-Octen-3-one (rOAV = 1.08 × 10^5^), characterized by its typical mushroom-like aroma; *β*-Damascone, (rOAV = 3.89 × 10^4^), imparting fruity and floral notes; Ethanone, 1-(2-aminophenyl)- (rOAV = 1.16 × 10^4^), contributing sweet aromas; 3(2H)-Furanone, dihydro-2-methyl- (rOAV = 6.22 × 10^5^), associated with buttery and nutty notes; and 1,3,5-Trithiane (rOAV = 9.57 × 10^3^), contributing sulfurous notes, exhibited markedly higher average rOAVs compared to their wild counterparts. In contrast, the seven wild species displayed higher average rOAVs for compounds including 3-Octen-2-one (rOAV = 4.23 × 10^3^), contributing grassy notes; the green and cucumber-like (E,Z)-2,6-Nonadienal (rOAV = 1.89 × 10^4^), (Z,Z)-3,6-Nonadienal (rOAV = 3.27 × 10^3^), and 2,4-Nonadienal (rOAV = 3.25 × 10^4^); (5Z)-Octa-1,5-dien-3-one (rOAV = 2.13 × 10^4^), associated with geranium-like notes; Benzenemethanethiol (rOAV = 5.17 × 10^3^), contributing alliaceous aromas; Non-8-enal (rOAV = 2.71 × 10^3^), associated with smoky notes; and Pyrazine, 2-ethyl-3,5-dimethyl- (rOAV = 1.44 × 10^4^), contributing roasty notes. This finding indicates a clear divergence in flavor characteristics between cultivated and wild *Morchella* species.

### 3.3. Correlation Analysis Among Geographical Origin, Taste-Active Amino Acids, and Core VOCs in Morchella

#### 3.3.1. Correlation Analysis Between Taste-Active Amino Acids and the rOAVs of Key VOCs

**Figure 3:**
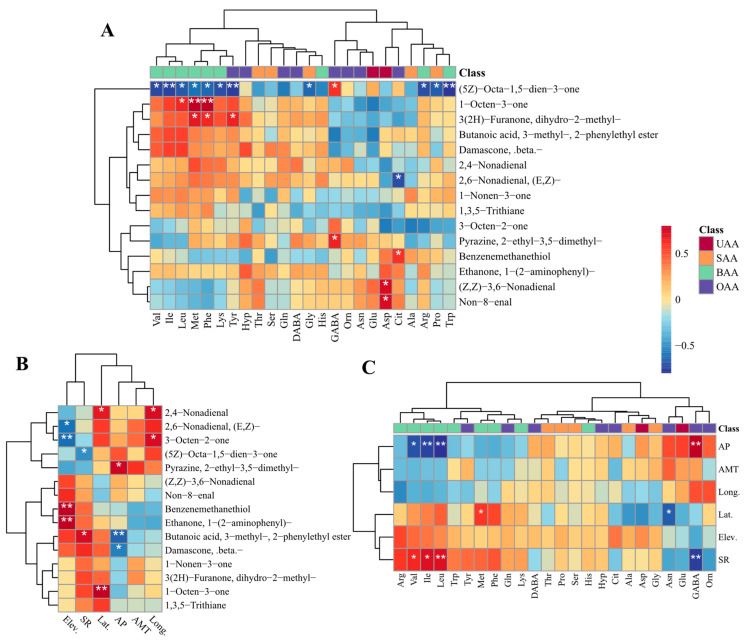
Correlation analysis between taste-active amino acids and the rOAVs of core VOCs (**A**); correlation analysis between environmental factors and the rOAVs of core VOCs (**B**) and taste-active amino acids (**C**); and * *p* < 0.05 and ** *p* < 0.01.

To investigate the intrinsic metabolic associations between taste-active amino acids and core volatile organic compounds across the 12 *Morchella* species from different species and geographical origins, Spearman’s correlation analysis was performed on the 24 detected taste-active amino acids and the 15 VOCs with rOAV > 1000. The results are presented as a heatmap (Figure 3). The analysis revealed a complex correlation network between key aroma-contributing ketones, aldehydes, alcohols, and heterocyclic compounds and free amino acids with distinct taste characteristics (umami, sweet, bitter, and tasteless). Notably, the C8 ketone (5Z)-Octa-1,5-dien-3-one exhibited the most extensive correlation pattern, demonstrating significant negative correlations with 11 amino acids (*p* < 0.05). Particularly strong negative correlations were observed with the bitter amino acids Ile, Tyr, and Trp (*p* < 0.01). Conversely, this compound showed a significant positive correlation exclusively with GABA (*p* < 0.05). Additionally, GABA exhibited a significant positive correlation with the pyrazine compound Pyrazine, 2-ethyl-3,5-dimethyl- (*p* < 0.05). The mushroom-characteristic VOCs 1-Octen-3-one showed highly significant positive correlations with the bitter amino acids Met and Phe (*p* < 0.01). Furthermore, 3(2H)-Furanone, dihydro-2-methyl- demonstrated significant positive correlations with Met, Phe, and Tyr (*p* < 0.05). Notably, Asp (UAA) exhibited significant positive correlations with the aldehydes (Z,Z)-3,6-Nonadienal and Non-8-enal (*p* < 0.05). The amino acid Cit showed a significant positive correlation with Benzenemethanethiol (*p* < 0.05) and a significant negative correlation with (E,Z)-2,6-Nonadienal (*p* < 0.05). These findings suggest that the flavor expression in different *Morchella* species may involve both synergistic enhancement and inhibitory attenuation effects between taste and aroma components. This provides a theoretical foundation for subsequent investigations into the mechanistic basis of flavor differentiation among *Morchella* species.

#### 3.3.2. Correlation Analysis Between Environmental Factors and Taste-Active Amino Acids and the rOAVs of Key VOCs

To investigate the influence of geographical environmental factors on the flavor quality formation of *Morchella*, Spearman correlation analysis was performed between geographical parameters—including Long., Lat., Elev., AMT, AP, and SR—at the collection sites of different *Morchella* species and the content of taste-active amino acids as well as the rOAVs of VOCs. The results are presented in Figure 3B,C. Long. exhibited significant positive correlations with 2,4-Nonadienal and 3-Octen-2-one (*p* < 0.05). Lat. showed significant positive correlations with 2,4-Nonadienal and Met (*p* < 0.05), a highly significant positive correlation with the characteristic fungal flavor compound 1-Octen-3-one (*p* < 0.01), and a significant negative correlation with Asn (*p* < 0.05). Elev. demonstrated highly significant positive correlations with Benzenemethanethiol and Ethanone, 1-(2-aminophenyl)- (*p* < 0.01), a significant negative correlation with (E,Z)-2,6-Nonadienal (*p* < 0.05), and a highly significant negative correlation with 3-Octen-2-one (*p* < 0.01). AP exhibited significant negative correlations with β-Damascone and Val (*p* < 0.05), highly significant negative correlations with Butanoic acid, 3-methyl-, 2-phenylethyl ester and the bitter amino acids Ile and Leu (*p* < 0.01), and significant positive correlations with Pyrazine, 2-ethyl-3,5-dimethyl- and GABA (*p* < 0.05). SR showed a significant negative correlation with (5Z)-Octa-1,5-dien-3-one (*p* < 0.05), a highly significant negative correlation with GABA (*p* < 0.01), and significant positive correlations with Butanoic acid, 3-methyl-, 2-phenylethyl ester and the bitter amino acids Val, Ile, and Leu (*p* < 0.05). These results demonstrate extensive associations between environmental factors and the formation of characteristic flavor profiles in *Morchella*, indicating that geographical and climatic conditions play a substantial role in shaping the metabolic pathways responsible for both taste-active amino acid accumulation and volatile organic compound biosynthesis.

## 4. Discussion

### 4.1. Characterization of Taste-Active Amino Acid Composition and Varietal Differences in Morchella

In this study, Gln and Arg were notably identified as the most abundant amino acids across all *Morchella* species. Although Gln is classified as a tasteless amino acid without direct taste-active properties, it serves as a biosynthetic precursor for Glu (UAA). Through enzymatic or thermal conversion during processing or consumption, Gln can be transformed into Glu, thereby contributing to umami perception [55]. Arg, despite being categorized as a bitter amino acid, possesses a relatively high taste threshold and can participate in Kokumi effects when interacting with other flavor compounds, enhancing the complexity, fullness, and lingering perception of overall flavor [56,57]. The high abundance of both amino acids contributes to the complex and robust flavor foundation of morels, corroborating the findings from our VOCs analysis. Furthermore, the observation that the five cultivated *Morchella* species exhibited higher average total amino acid content compared to the seven wild species may be attributed to differences in growth conditions. Unlike the complex and variable natural environment, controlled cultivation provides relatively stable growth conditions (temperature, humidity, and light) and abundant nutritional resources (carbon and nitrogen sources). This stable environment not only supplies ample substrates for amino acid biosynthesis but also potentially redirects metabolic resources from stress-responsive pathways—such as the synthesis of protective compounds like heat shock proteins and trehalose—toward primary metabolism, including protein and amino acid synthesis, thereby resulting in elevated total amino acid accumulation [58]. Notably, beyond their nutritional significance, amino acids also function as precursors for volatile organic compounds. The observed differences in amino acid composition and content among various *Morchella* species therefore establish a fundamental basis for their distinct overall flavor profiles.

In food sensory science, the taste characteristics of amino acids are primarily determined by their side chain structures and receptor recognition mechanisms. BAAs typically possess hydrophobic side chains, and their bitterness perception arises from the interaction between these hydrophobic groups and bitter taste receptors (T2Rs) in the oral cavity [26]. Within an appropriate concentration range, BAA can enhance flavor complexity and layering; however, excessive levels may lead to decreased sensory acceptability [59]. SAA elicit sweet taste perception through activation of sweet taste receptors (T1R2/T1R3) [45], while UAA contribute umami perception via activation of umami receptors (T1R1/T1R3) and synergistically interact with Kokumi compounds to enrich the overall flavor profile of foods [27,60]. Based on the proportions of UAA, SAA, and BAA in the amino acid profiles of *Morchella* and their corresponding TAVs, the investigated species were categorized into three distinct flavor types: bitterness-dominant (XHQH-2 and XZ), sweet-umami synergistic (XGLL and GN), and bitter-umami complex (GL). The high proportion and elevated TAVs of BAA observed in XHQH-2 and XZ may be attributed to their elevated protease activity or specific expression patterns of amino acid transporters [61,62]. Importantly, the bitterness-dominant classification does not necessarily imply a negative flavor perception for XHQH-2 and XZ. On the contrary, BAA such as Leu, Val, Ile, and Phe serve as key precursors for Strecker degradation reactions [63], playing a crucial role in the generation of characteristic volatile organic compounds. This may constitute the material basis for the intense fungal aroma characteristic of XHQH-2 and XZ. In contrast, XGLL and GN exhibited enhanced palatability, aligning with consumer preferences for edible fungi sensory quality—characterized by clean, sweet, and fresh taste with harmonious flavor layering upon fresh consumption. This positions these species as having considerable potential for development into ready-to-eat or freeze-dried products [64,65]. Furthermore, the complementary interplay between bitterness and umami in GL results in a more complex and distinctive flavor profile, rendering it suitable for development as a flavoring or seasoning product [66].

### 4.2. Comparative Analysis of VOCs Between Cultivated and Wild Morchella Species

This study revealed that cultivated *Morchella* species exhibited VOCs with substantial rOAV contributions characterized predominantly by fruity, sweet, and buttery notes, while retaining the typical mushroom aroma. We hypothesize that this flavor profile may be associated with the composition and proportion of nutrients in the cultivation substrate used for artificial morel production. Previous research has demonstrated that the flavor quality of *Lentinula edodes* is influenced by the types and ratios of carbon and nitrogen sources in the cultivation medium, which subsequently affect the content of taste-active amino acids and soluble sugars, ultimately resulting in distinct flavor characteristics [67]. In contrast, wild *Morchella* species displayed VOCs with rOAV contributions predominantly characterized by green, alliaceous, and smoky notes. This distinctive profile may be attributable to the natural growth environment stimulating metabolic processes in the fruiting bodies, thereby influencing flavor development. Motýlová et al. reported that environmental stressors—including temperature fluctuations, humidity variations, and microbial interactions—induce edible fungi to produce diverse secondary metabolites, including sulfur-containing compounds and ketones, as adaptive responses to challenging environmental conditions [68]. The biosynthesis of these metabolites involves fatty acid oxygenases in fatty acid metabolism and the expression of stress-responsive genes such as HSP20 and those encoding P450 enzymes in sulfur-containing compound metabolism [69,70]. These metabolic adaptations collectively contribute to the more pronounced wild, grassy-smoky composite flavor characteristics observed in wild morels. Collectively, the flavor profile of cultivated *Morchella* species—characterized by harmonious integration of fruity and sweet notes with the fundamental mushroom taste—aligns closely with mainstream consumer preferences, rendering these species particularly suitable for fresh consumption and food product development. In contrast, the more intense and complex flavor profile of wild species offers distinctive sensory characteristics that appeal to consumers seeking “wild” flavor experiences and positions them favorably for the premium culinary market segment.

### 4.3. Elucidation of Metabolic Associations Between Taste-Active Amino Acids and Core VOCs and the Underlying Flavor Formation Mechanism in Morchella

#### 4.3.1. Correlation Analysis Between C8 Ketone VOCs and Taste-Active Amino Acids

C8 compounds serve as characteristic flavor constituents in most edible fungi, with their biosynthesis primarily originating from the lipoxygenase (LOX) pathway of fatty acid metabolism. This pathway generates C8 compounds using linoleic acid 10-hydroperoxide as a substrate—a metabolic route unique to fungi and constituting the molecular basis that distinguishes the “fresh mushroom” aroma of edible fungi from the volatile profiles of plant-derived foods. In the present study, two typical C8 compounds were identified: (5Z)-Octa-1,5-dien-3-one and 1-Octen-3-one. Notably, these two compounds exhibited completely divergent correlation patterns with taste-active amino acids. The geranium-like (5Z)-Octa-1,5-dien-3-one demonstrated negative correlations with the majority of BAA. It is hypothesized that this inverse relationship may arise from substrate competition between amino acid biosynthesis and the LOX pathway. When metabolic flux is preferentially directed toward the LOX pathway, the allocation of substrates for amino acid synthesis may be correspondingly suppressed, manifesting as negative correlations [71]. This observation also suggests that substantial accumulation of BAA in *Morchella* fruiting bodies may attenuate the contribution of geranium-like notes to the overall flavor profile. Conversely, the mushroom-like 1-Octen-3-one exhibited positive correlations with Leu, Met, and Phe. This finding suggests that these amino acids may function as signaling molecules modulating enzyme activities within the LOX pathway, thereby promoting the generation of 1-Octen-3-one [72]. Furthermore, this indicates that *Morchella* species characterized by elevated concentrations of Leu, Met, and Phe tend to exhibit more pronounced mushroom aroma profiles. Notably, these amino acids possess relatively high taste thresholds (range: 0.30~1.90 mg/g), rendering their bitterness perception less prominent. Instead, through Kokumi effects, they may contribute to enriching the overall flavor complexity of morels without imparting undesirable bitter notes.

#### 4.3.2. Correlation Analysis Between Aromatic and Heterocyclic Compound VOCs and Taste-Active Amino Acids

Benzenemethanethiol, characterized by its intense alliaceous aroma, is typically generated through Strecker degradation of Phe followed by thiolation, or alternatively through direct incorporation of sulfur moieties derived from Met [73,74]. However, in the present study, Benzenemethanethiol exhibited a significant positive correlation with Cit, an intermediate metabolite in nitrogen metabolism. It is hypothesized that samples with elevated Cit accumulation may possess more active nitrogen metabolism and consequently enhanced sulfur moiety availability, thereby facilitating the generation of Benzenemethanethiol. Conversely, Cit demonstrated a negative correlation with the cucumber-like C9 aldehyde compound (E,Z)-2,6-Nonadienal, suggesting a potential antagonistic relationship between nitrogen metabolism and fatty acid metabolism [75]. When nitrogen metabolism is highly active, the biosynthetic potential for sulfur-containing VOCs appears enhanced, while the synthesis of cucumber-like VOCs derived from fatty acid metabolism may be correspondingly suppressed. This observation further suggests that Cit content may function as a potential indicator for assessing the sulfurous and green aroma characteristics of *Morchella*. However, elucidation of the underlying regulatory mechanisms will require subsequent integrated analysis incorporating metabolomic and transcriptomic approaches.

Pyrazine, 2-ethyl-3,5-dimethyl-, a pyrazine compound characterized by typical roasty, nutty, and coffee-like cocoa aromas, is typically generated through the Maillard reaction or Strecker degradation involving amino acids and reducing sugars [76]. GABA, a non-proteinogenic amino acid, has been demonstrated in previous studies to enhance the membrane integrity of Agaricus bisporus and delay the lignification process in its fruiting bodies [77]. This suggests that GABA accumulation is closely associated with the physiological status of fungi, including factors such as maturation stage and postharvest storage duration. The positive correlation observed between GABA and this pyrazine compound suggests that the rOAV expression of pyrazine compounds may be linked to the developmental stage and physiological state of *Morchella*. This finding provides an important direction for subsequent investigations: achieving enhanced roasty and nutty aroma characteristics in the overall flavor profile of morels may be achievable through strategic regulation of harvest timing based on the growth stage of the fruiting bodies.

#### 4.3.3. Correlation Analysis Between Aldehyde VOCs and Taste-Amino Acids

In addition to the previously discussed (E,Z)-2,6-Nonadienal, the present study revealed that the remaining two C9 aldehyde compounds—Non-8-enal and (Z,Z)-3,6-Nonadienal—exhibited positive correlations with Asp. This phenomenon may be attributed to the metabolic role of Asp as an intermediate in the tricarboxylic acid (TCA) cycle. Elevated Asp content suggests enhanced TCA cycle flux, thereby providing sufficient ATP to support fatty acid metabolism. Notably, both of these C9 aldehydes are oxidation degradation products of polyunsaturated fatty acids such as linoleic acid [38,78]. From a flavor chemistry perspective, Non-8-enal contributes smoky notes, while (Z,Z)-3,6-Nonadienal imparts fatty and cucumber-like aromas. Asp, as a biosynthetic precursor for the umami amino acid Glu, directly influences umami intensity through its abundance [71]. Therefore, the positive correlation observed between Asp and these two aldehydes elucidates a synergistic mechanism at the metabolic level between taste substances and aroma compounds. It is hypothesized that in species characterized by high Asp content (e.g., XGLL), not only is umami perception prominently expressed, but the expression of smoky and fatty notes is also correspondingly enhanced. This coordinated metabolic interplay contributes to the formation of superior flavor characteristics characterized by coherent “umami-aroma harmony.”

### 4.4. Regulatory Mechanisms of Geographical Environmental Factors on Taste-Active Amino Acids and Key VOCs in Morchella

To systematically elucidate the formation mechanism of geographically characteristic flavors in *Morchella*, understanding the relationship between environmental factors and flavor compounds is of paramount importance. Elev. as a critical geographical environmental factor, can influence microbial community structure and function through ultraviolet radiation, temperature, and soil physicochemical properties [6]. In the present study, the negative correlations between Elev. and both 3-Octen-2-one and (E,Z)-2,6-Nonadienal suggest that high altitude may suppress the LOX pathway, thereby reducing the biosynthesis of aldehyde and ketone VOCs. Benzenemethanethiol, a sulfur-containing VOC typically induced in fungi under environmental stress [79], exhibited a positive correlation with Elev., indicating that low-temperature and hypoxic conditions at high altitudes may activate sulfur metabolic pathways in *Morchella*, thereby promoting the accumulation of defensive secondary metabolites such as Benzenemethanethiol. Similarly, SR showed a negative correlation with GABA, a stress-responsive signaling molecule, suggesting that intense SR may accelerate GABA catabolism, leading to reduced GABA content [80]. Conversely, the bitter amino acids Val, Ile, and Leu exhibited positive correlations with SR, potentially attributable to photo-stress inducing the accumulation of these primary metabolites in *Morchella*. Variations in Long. and Lat. are typically accompanied by climatic differences, including substantial variations in temperature, photoperiod, precipitation, and evaporation [81]. Our findings revealed positive correlations between Long., Lat., and the cucumber-like aldehyde 2,4-Nonadienal. This may be attributed to the influence of Long. and Lat. on the expression of LOX pathway-related enzymes or genes, thereby promoting the accumulation of these VOCs. Notably, AP exhibited negative correlations with Butanoic acid, 3-methyl-, 2-phenylethyl ester, β-Damascone, Val, Ile, and Leu, suggesting that high AP may induce root hypoxia or soil nutrient leaching in *Morchella*, thereby inhibiting the synthesis and accumulation of taste-active amino acids and VOCs [82]. In contrast, GABA accumulation was enhanced under high AP conditions, further corroborating that fungi accumulate the stress-responsive signaling molecule GABA under hypoxic stress to adapt to environmental changes. These findings indicate that climatic and environmental factors may influence the overall flavor expression of *Morchella* through regulation of primary metabolism (amino acid biosynthesis) and secondary metabolism (VOCs transformation). Furthermore, the relationships between soil physicochemical properties—including pH, organic matter content, total nitrogen, total phosphorus, total potassium, and cation exchange capacity—and *Morchella* flavor compounds warrant further investigation.

In summary, integrating the composition and proportions of free amino acids, the TAV of taste-active amino acids, the rOAV of volatile organic compounds, and their correlation with environmental factors, the flavor characteristics and potential application directions of the 12 *Morchella* species were systematically categorized into two major flavor groups and seven subgroups. Group I (Cultivated species: XHQH-2, XHQH-6, JZ, TJ, PA) is characterized by fruity-sweet and mushroom-like notes with a mellow flavor, making them suitable for fresh consumption. Subgroup 1 (JZ, PA) exhibits the most prominent sweet-mushroom flavor, ideal for freeze-dried products and beverages. Subgroup 2 (XHQH-6, TJ) features additional alliaceous notes, suitable for aroma-enhanced products. Subgroup 3 (XHQH-2) possesses subtle bitterness with enhanced flavor layering, suitable for mushroom-based seasonings. Group II (Wild species: XZ, QL, CBS, XGLL, HZ, GL, GN) is characterized by grassy and smoky notes with complex and intense flavor, suitable for composite food products. Subgroup 4 (XGLL, GN) exhibits supplementary sweet aromas, also suitable for fresh consumption. Subgroup 5 (XZ, GL) displays subtle bitterness, suitable for functional foods (e.g., oral liquids). Subgroup 6 (CBS, QL) features pronounced roasty notes, suitable for thermal processing or fermented products (e.g., *Morchella* soy sauce). Subgroup 7 (HZ) exhibits subdued wild characteristics, suitable for soup applications.

## 5. Conclusions

This study systematically compared the amino acid profiles and volatile flavor characteristics of 12 *Morchella* species from different species and geographical origins. The results revealed that the five cultivated species (XHQH-2, XHQH-6, JZ, TJ, PA) exhibited higher average total amino acid contents and prominent volatile compounds including 1-Octen-3-one, β-Damascone, and Ethanone, 1-(2-aminophenyl)-, resulting in harmonious fruity-sweet and mushroom-like flavor profiles suitable for fresh consumption and lightly processed products. In contrast, the seven wild species (XZ, QL, CBS, XGLL, HZ, GL, GN) were characterized by dominant volatile compounds such as (E,Z)-2,6-Nonadienal, Benzenemethanethiol, and Non-8-enal, contributing to complex grassy and smoky flavor profiles suitable for high-end composite food development. Correlation analysis elucidated the flavor formation mechanisms involving geographical environmental factors, taste-active amino acids, and the rOAVs of core VOCs. For instance, Leu, Met, and Phe may regulate the expression of 1-Octen-3-one through the LOX metabolic pathway; Asp can influence linoleic acid metabolism via the TCA cycle, thereby indirectly modulating the expression of Non-8-enal and (Z,Z)-3,6-Nonadienal; and environmental factors including Long., Lat., Elev., AP, and SR can regulate the characteristic flavor profiles of *Morchella* by influencing both primary and secondary metabolism. These findings provide insights into the mechanistic basis of flavor differentiation among *Morchella* species and establish a scientific foundation for their differential development and utilization. Limitations and Future Perspectives: This study focused exclusively on free amino acids among non-volatile taste compounds. Subsequent investigations should quantify the 5′-nucleotide content and calculate the equivalent umami concentration (EUC) to achieve more comprehensive characterization of umami taste attributes in *Morchella*. Furthermore, integration of multi-omics approaches—including metabolomics and sensomics—will facilitate elucidation of the molecular mechanisms underlying flavor differences among *Morchella* species, ultimately enabling directed flavor regulation.

## Figures and Tables

**Table 1:** Geographical conditions of morel mushroom production areas ^1^.

Sample	Species	Long. ^2^	Lat. ^3^	Elev. ^4^	AMT ^5^	AP ^6^	SR ^7^
XHQH-2	*M. septimelata*	100.15	37.33	3317	0.02	381	16,147
XHQH-6	*M. septimelata*	100.15	37.33	3317	0.02	381	16,147
JZ	*M. sextelata*	102.04	35.94	2028	7.55	406	15,266
TJ	*M. sextelata*	99.02	37.30	3408	−0.59	273	16,586
PA	*M. sextelata*	102.11	36.50	2104	6.98	358	15,498
XZ	Wild	94.36	29.65	2991	8.91	664	15,919
QL	Wild	109.71	34.10	1495	9.13	736	14,120
CBS	Wild	128.43	42.22	1222	0.85	727	13,197
XGLL	Wild	99.74	27.84	3311	6.56	703	14,263
HZ	Wild	101.96	36.84	2528	3.89	409	15,524
GL	Wild	100.24	34.47	3730	−0.20	522	14,909
GN	Wild	102.91	34.98	2903	3.20	565	14,315

^1^ Climate data were obtained from the WorldClim 2.1 database (https://worldclim.org). ^2^ Long.: Longitude (°E). ^3^ Lat.: Latitude (°N). ^4^ Elev.: Elevation (m). ^5^ AMT: Annual mean temperature (°C). ^6^ AP: Annual precipitation (mm). ^7^ SR: Solar radiation (corresponding to solar irradiance in climatology, with the unit kJ m^−2^ day^−1^, data values have been rounded to integers).

**Table 2:** TAV of taste-active amino acids in different species of *Morchella*.

Free Amino Acids (mg/g)	Threshold (mg/g)	TAV											
		XHQH-2	XHQH-6	JZ	TJ	PA	XZ	QL	CBS	XGLL	HZ	GL	GN
UAA													
Asp	1.00	0.246 ± 0.056	0.40 ± 0.12	0.338 ± 0.068	0.189 ± 0.037	0.123 ± 0.026	0.613 ± 0.092	0.287 ± 0.015	0.1774 ± 0.0042	0.250 ± 0.020	0.265 ± 0.058	0.87 ± 0.17	0.573 ± 0.048
Glu	0.30	3.53 ± 0.66	7.5 ± 1.9	5.76 ± 0.35	4.29 ± 0.76	7.8 ± 1.3	11.59 ± 0.79	10.0 ± 1.9	9.45 ± 0.23	6.01 ± 0.48	10.45 ± 0.54	13.51 ± 0.39	7.8 ± 1.8
SAA													
Pro	3.00	0.160 ± 0.069	0.172 ± 0.069	0.301 ± 0.064	0.318 ± 0.070	0.143 ± 0.047	0.2806 ± 0.0061	0.296 ± 0.087	0.205 ± 0.049	0.121 ± 0.012	0.1433 ± 0.0017	0.3856 ± 0.0060	0.149 ± 0.021
Ala	0.60	2.00 ± 0.43	3.6 ± 1.0	9.21 ± 0.72	7.1 ± 1.4	5.08 ± 0.93	4.97 ± 0.46	4.41 ± 0.75	3.55 ± 0.18	5.78 ± 0.28	3.494 ± 0.042	6.73 ± 0.31	4.8 ± 0.9
Thr	2.60	0.234 ± 0.028	0.308 ± 0.063	0.380 ± 0.016	0.207 ± 0.024	0.157 ± 0.018	0.302 ± 0.057	0.372 ± 0.031	0.2145 ± 0.0057	0.164 ± 0.020	0.227 ± 0.026	0.716 ± 0.080	0.276 ± 0.019
Gly	1.30	0.307 ± 0.024	0.318 ± 0.047	1.12 ± 0.24	0.302 ± 0.021	0.239 ± 0.026	0.34 ± 0.11	0.422 ± 0.086	0.254 ± 0.011	0.304 ± 0.047	0.290 ± 0.088	0.438 ± 0.072	0.177 ± 0.033
Ser	1.50	0.670 ± 0.080	0.61 ± 0.12	1.39 ± 0.21	0.580 ± 0.072	0.401 ± 0.054	0.481 ± 0.054	0.68 ± 0.10	0.561 ± 0.013	0.644 ± 0.084	0.85 ± 0.18	1.16 ± 0.21	0.369 ± 0.063
BAA													
Arg	0.50	22.3 ± 1.7	17.68 ± 0.93	20.9 ± 2.2	24.92 ± 0.39	6.49 ± 0.43	31.0 ± 6.2	12.02 ± 0.25	7.57 ± 0.82	1.82 ± 0.38	16.0 ± 2.1	32.6 ± 4.5	3.90 ± 0.12
Lys	0.50	2.27 ± 0.30	1.56 ± 0.37	4.148 ± 0.032	1.78 ± 0.26	1.04 ± 0.13	1.47 ± 0.23	2.24 ± 0.34	1.14 ± 0.14	0.341 ± 0.051	0.86 ± 0.16	1.94 ± 0.36	0.525 ± 0.012
His	0.20	4.319 ± 0.087	2.420 ± 0.034	5.00 ± 0.50	2.126 ± 0.036	1.802 ± 0.042	3.16 ± 0.81	3.82 ± 0.25	2.21 ± 0.22	0.76 ± 0.16	2.42 ± 0.52	7.5 ± 1.3	1.30 ± 0.25
Val	0.40	1.21 ± 0.28	1.31 ± 0.37	2.26 ± 0.22	1.69 ± 0.38	1.03 ± 0.23	1.01 ± 0.15	0.713 ± 0.075	0.625 ± 0.058	0.517 ± 0.014	0.855 ± 0.055	1.59 ± 0.19	0.424 ± 0.034
Phe	0.90	0.4553 ± 0.0071	0.2598 ± 0.026	0.311 ± 0.039	0.2852 ± 0.0073	0.1016 ± 0.0058	0.201 ± 0.023	0.173 ± 0.028	0.2210 ± 0.0087	0.0750 ± 0.012	0.0926 ± 0.0061	0.164 ± 0.016	0.06553 ± 0.00046
Ile	0.90	0.426 ± 0.049	0.402 ± 0.075	0.622 ± 0.029	0.496 ± 0.051	0.321 ± 0.043	0.260 ± 0.049	0.1707 ± 0.0098	0.168 ± 0.012	0.088 ± 0.011	0.255 ± 0.046	0.334 ± 0.064	0.0760 ± 0.011
Met	0.30	0.106 ± 0.029	0.094 ± 0.025	0.0939 ± 0.0041	0.078 ± 0.022	0.0093 ± 0.0018	0.0356 ± 0.0064	0.0642 ± 0.0099	0.0687 ± 0.0091	0.00364 ± 0.00064	0.0070 ± 0.0021	0.0116 ± 0.0047	0.0041 ± 0.0016
Trp	0.90	0.0659 ± 0.0020	0.0959 ± 0.0050	0.207 ± 0.026	0.1592 ± 0.0031	0.0836 ± 0.0027	0.115 ± 0.020	0.100 ± 0.017	0.0576 ± 0.0031	0.01537 ± 0.0018	0.101 ± 0.013	0.159 ± 0.023	0.0369 ± 0.0071
Leu	1.90	0.196 ± 0.043	0.195 ± 0.046	0.274 ± 0.041	0.237 ± 0.047	0.133 ± 0.025	0.116 ± 0.012	0.0897 ± 0.0056	0.1010 ± 0.0079	0.03767 ± 0.0012	0.103 ± 0.010	0.126 ± 0.020	0.02260 ± 0.0033
OAA													
Tyr	0.91	0.723 ± 0.023	0.466 ± 0.051	0.870 ± 0.062	0.340 ± 0.015	0.323 ± 0.024	0.77 ± 0.11	0.416 ± 0.040	0.2572 ± 0.0074	0.165 ± 0.028	0.239 ± 0.050	0.362 ± 0.071	0.161 ± 0.014
Asn	1.00	1.078 ± 0.012	1.99 ± 0.22	4.43 ± 0.27	1.038 ± 0.038	1.718 ± 0.054	2.51 ± 0.46	4.23 ± 0.11	1.75 ± 0.19	2.50 ± 0.49	2.27 ± 0.27	7.9 ± 1.0	1.752 ± 0.040
GABA	- ^1^	-	-	-	-	-	-	-	-	-	-	-	-
Orn	-	-	-	-	-	-	-	-	-	-	-	-	-
Cit	-	-	-	-	-	-	-	-	-	-	-	-	-
DABA	-	-	-	-	-	-	-	-	-	-	-	-	-
Hyp	-	-	-	-	-	-	-	-	-	-	-	-	-
Gln	3.00	2.85 ± 0.22	4.56 ± 0.13	6.46 ± 0.91	3.70 ± 0.28	2.25 ± 0.15	1.77 ± 0.34	4.41 ± 0.32	1.94 ± 0.23	0.62 ± 0.12	2.89 ± 0.50	7.3 ± 1.4	1.364 ± 0.018

^1^—denotes the taste threshold.

**Table 3:** rOAV of key VOCs in different *Morchella* species (rOAV > 1000).

Compounds	Odor ^1^	Threshold (μg/g) ^2^	rOAV											
			XHQH-2	XHQH-6	JZ	TJ	PA	XZ	QL	CBS	XGLL	HZ	GL	GN
3-Octen-2-one	earthy, herbal, sweet, mushroom, blueberry	3.00 × 10^−5^	2649 ± 84	3339 ± 73	2291 ± 47	1430.7 ± 6.4	7999 ± 92	1570 ± 110	5130 ± 100	14,000 ± 1000	2230 ± 120	3211 ± 96	1125 ± 33	2010 ± 160
2,6-Nonadienal, (E,Z)-	cucumber, green	1.00 × 10^−5^	14,210 ± 760	14,180 ± 920	29,600 ± 3700	9860 ± 640	16,260 ± 610	13,800 ± 1800	14,400 ± 1000	60,000 ± 5000	8900 ± 360	14,680 ± 560	11,650 ± 360	8290 ± 590
Butanoic acid, 3-methyl-, 2-phenylethyl ester	floral, fruity, sweet	1.00 × 10^−5^	2920 ± 650	2530 ± 260	1900 ± 310	1856 ± 92	2300 ± 110	1610 ± 140	1224 ± 57	1091 ± 49	1490 ± 120	1480 ± 150	1810 ± 75	1901 ± 53
(Z,Z)-3,6-Nonadienal	fatty, cucumber	5.00 × 10^−5^	3320 ± 170	3370 ± 120	2930 ± 190	2860 ± 120	3190 ± 190	3450 ± 170	2957 ± 74	2880 ± 150	3220 ± 140	3230 ± 140	3550 ± 130	3600 ± 200
1-Nonen-3-one	mushroom	1.00 × 10^−6^	15,600 ± 3800	79,200 ± 4500	30,000 ± 10,000	41,000 ± 3000	38,100 ± 5900	40,000 ± 10,000	28,500 ± 2300	27,200 ± 1400	23,050 ± 690	21,870 ± 680	20,000 ± 4000	24,000 ± 1900
(5Z)-Octa-1,5-dien-3-one	geranium	3.00 × 10^−6^	9350 ± 440	9000 ± 760	5856 ± 33	4980 ± 320	11,800 ± 800	7720 ± 120	8270 ± 450	75,500 ± 2600	24,150 ± 820	10,980 ± 410	10,220 ± 780	12,060 ± 810
1-Octen-3-one	mushroom	5.00 × 10^−6^	176,400 ± 2900	256,300 ± 6900	58,900 ± 1800	30,000 ± 1600	20,500 ± 1600	17,000 ± 1900	16,900 ± 1500	61,400 ± 3700	12,960 ± 510	15,000 ± 1000	8150 ± 420	11,000 ± 1600
Damascone, .beta.-	floral, berry, tobacco	2.00 × 10^−6^	84,500 ± 9700	59,000 ± 15,000	23,600 ± 3400	7010 ± 460	20,300 ± 1100	6330 ± 710	3580 ± 290	5010 ± 420	15,600 ± 1400	21,040 ± 710	15,800 ± 1200	8680 ± 340
Ethanone, 1-(2-aminophenyl)-	grape, sweet	2.70 × 10^−4^	19,850 ± 450	18,240 ± 810	7880 ± 840	7150 ± 230	5120 ± 450	11,070 ± 460	4444 ± 70	6305 ± 68	11,300 ± 400	8000 ± 160	18,410 ± 290	11,070 ± 530
3(2H)-Furanone, dihydro-2-methyl-	sweet, buttery, nutty	5.00 × 10^−6^	(9.01 ± 0.23) × 10^5^	(7.04 ± 0.49) × 10^5^	(4.69 ± 0.18) × 10^5^	(4.14 ± 0.23) × 10^5^	(6.23 ± 0.24) × 10^5^	(5.25 ± 0.17) × 10^5^	700,700 ± 8600	(4.03 ± 0.17) × 10^5^	(3.67 ± 0.13) × 10^5^	(3.86 ± 0.17) × 10^5^	264,900 ± 3900	(2.92 ± 0.17) × 10^5^
Benzenemethanethiol	alliaceous, sulfury, coffee	3.50 × 10^−6^	4960 ± 250	5390 ± 230	4110 ± 230	5860 ± 390	5220 ± 530	5351 ± 82	4740 ± 190	4180 ± 120	5300 ± 2100	4290 ± 380	5670 ± 240	6610 ± 990
Non-8-enal	smoky	2.00 × 10^−4^	2790 ± 130	2800 ± 100	2460 ± 150	2378 ± 89	2610 ± 140	2860 ± 150	2464 ± 49	2350 ± 150	2681 ± 97	2657 ± 86	2970 ± 120	3000 ± 170
1,3,5-Trithiane	sulfury	4.00 × 10^−5^	6500 ± 600	7941 ± 94	9650 ± 940	18,350 ± 480	5390 ± 780	6710 ± 150	4830 ± 340	11,000 ± 500	7964 ± 88	10,780 ± 860	3600 ± 260	4930 ± 420
Pyrazine, 2-ethyl-3,5-dimethyl-	roasted, nutty, coffee	4.00 × 10^−5^	7590 ± 190	12,570 ± 260	10,600 ± 380	2110 ± 110	4883 ± 58	11,790 ± 360	32,258 ± 70	28,600 ± 1300	10,600 ± 400	7140 ± 140	3237 ± 42	7470 ± 310
2,4-Nonadienal	fatty, green, cucumber	5.00 × 10^−5^	2409 ± 47	2910 ± 160	2380 ± 140	1759 ± 54	3560 ± 40	1200 ± 100	7200 ± 1000	(2.13 ± 0.13) × 10^5^	1470 ± 40	1593 ± 21	1680 ± 54	1680 ± 160

^1^ Information regarding odor characterizations was retrieved from publicly accessible flavor and fragrance databases, including The Good Scents Company (http://www.thegoodscentscompany.com), Perflavory (http://perflavory.com/), and the Food Flavor Laboratory Database (http://foodflavorlab.cn/#/home). ^2^ Odor threshold values are cited from references [53,54]; The unit ‘µg/g’ indicates the concentration in aqueous solution (µg analyte/g water).

## Data Availability

The original contributions presented in this study are included in the article/Supplementary Materials. Further inquiries can be directed to the corresponding authors.

## Supplementary Materials

The following supporting information can be downloaded at https://www.mdpi.com/article/10.3390/foods15081424/s1: Table S1: Volatile compound composition and relative content in different species of *Morchella*; Table S2: VOCs with rOAV ≥ 1 and their aroma attributes in different species of *Morchella*; Table S3: Contents of free amino acids in different *Morchella* species.

## Abbreviations

The following abbreviations are used in this manuscript:

Asp	L-Aspartic acid
Glu	L-Glutamic acid
Pro	L-Proline
Ala	L-Alanine
Thr	L-Threonine
Gly	Glycine
Ser	L-Serine
Arg	L-Arginine
Lys	L-Lysine
His	L-Histidine
Val	L-Valine
Phe	L-Phenylalanine
Ile	L-Isoleucine
Met	L-Methionine
Trp	L-Tryptophan
Leu	L-Leucine
Tyr	L-Tyrosine
Asn	L-Asparagine
GABA	Gamma-Aminobutyric acid
Orn	L-Ornithine
Cit	L-Citrulline
DABA	L-2,4-diaminobutyric acid
Hyp	4-Hydroxy-L-Proline
Gln	L-Glutamine
Cys-Cys	L-Cystine
